# PSMA-1007 Uptake in Ganglia of the Sympathetic Trunk and Its Intra-individual Reproducibility

**DOI:** 10.1007/s11307-022-01784-4

**Published:** 2022-11-11

**Authors:** E. Mamlins, D. Schmitt, M. Beu, K. Mattes-György, J. M. Henke, C. Antke, E. Novruzov, J. Cardinale, J. Kirchner, G. Niegisch, J. P. Radtke, L. Schimmöller, P. Albers, G. Antoch, F. L. Giesel

**Affiliations:** 1 Department of Nuclear Medicine, Medical Faculty and University Hospital Duesseldorf, Heinrich-Heine-University Duesseldorf, Moorenstrasse 5, 40225 Duesseldorf, Germany; 2 Department of Diagnostic and Interventional Radiology, Medical Faculty and University Hospital Duesseldorf, Heinrich-Heine-University Duesseldorf, Duesseldorf, Germany; 3 Department of Urology, Medical Faculty and University Hospital Duesseldorf, Heinrich-Heine-University Duesseldorf, Duesseldorf, Germany

**Keywords:** PSMA-1007, F-18 PSMA, Ganglia, Sympathetic trunk, PET, Prostate cancer

## Abstract

**Aim/Purpose:**

^18^F-labeled PSMA ligands offer various advantages as PET tracers over ^68^Ga-labeled PSMA counterparts. Especially, an improved spatial resolution leads to improved detection rates of smaller prostate cancer (PCa) lesions. However, physiological PSMA uptake of ganglia of the sympathetic trunk can be quickly misinterpreted as possible PSMA-positive lymph node metastases. The aim of this retrospective study is to investigate [^18^F]PSMA-1007 uptake and its intra-individual reproducibility in ganglia of the sympathetic trunk.

**Methods:**

We retrospectively included 28 consecutive patients (median age 69 ± 9 with a range of 49–90) with biochemical recurrence of PCa who underwent [^18^F]PSMA-1007 PET/CT scan and, accordingly, a follow-up examination between August 2018 and August 2021. Cervical, coeliac, and sacral ganglia were identified on the iterative PET reconstructions and correlated with CT component. Tracer uptake of ganglia was determined by measuring SUV_max_ and SUV_mean_ values. Anatomical position of the ganglia in relation to adjacent vertebral bodies were noted. Statistical analyses were conducted using two-way repeated measures ANOVA and descriptive statistics.

**Results:**

The highest [^18^F]PSMA-1007 uptake was found in coeliac ganglia followed by cervical and sacral ganglia. The SUV_max_ in coeliac ganglia was 3.13 ± 0.85 (follow-up scan 3.11 ± 0.93), in cervical ganglia 2.73 ± 0.69 (follow-up scan 2.67 ± 0.74), and in sacral ganglia 1.67 ± 0.50 (follow-up scan 1.64 ± 0.52). The SUV_mean_ in coeliac ganglia was 2.28 ± 0.64 (follow-up scan 2.28 ± 0.66), in cervical ganglia 1.62 ± 0.43 (follow-up scan 1.61 ± 0.43) and in sacral ganglia 1.15 ± 0.33 (follow-up scan 1.12 ± 0.34). In a given ganglion station, there was no statistically significant difference of SUV_max_ or SUV_mean_ values between baseline and follow-up scans.

**Conclusions:**

The first systematically described physiological [^18^F]PSMA-1007 uptake in ganglia of the sympathetic trunk showed a low variability of SUV_max_ or SUV_mean_ and a good intra-individual reproducibility of [^18^F]PSMA-1007 uptake in follow-up scans. These findings might improve and guide the differentiation of ganglia from possible malignant lesions.

## Introduction

The prostate-specific membrane antigen (PSMA) is a type II membrane protein which is expressed in normal prostate tissue as well as in prostate cancer (PCa) and its metastases [1, 2]. Therefore, it is a suitable target for molecular diagnostic imaging and therapeutic approaches in the context of PCa. Several PSMA ligands have been developed in the last decade. The ^68^Ga-labeled PSMA-ligand [^68^Ga]GaPSMA-11 introduced in 2011 is characterized by high sensitivity in detection of metastases of PCa and is widely used in Asia and Europe [3, 4]. However, currently, Gallium-68 still has a major limitation with respect to large-scale production: The nuclide is usually provided by commercial ^68^Ge/^68^Ga-generators limited to a capacity of maximum 1.8 GBq (before radiosynthesis of the ligand). Alternative cyclotron-based large-scale productions have been developed, but are still not commonly established [5, 6]. On the other hand, large-scale productions with fluorine-18 are commonly routine (e.g., for production of [^18^F]FDG); thus, research for ^18^F-labeled PSMA-ligands was also conducted even before [^68^Ga]Ga-PSMA-11 proved the clinical potential of this class of ligands.

[^18^F]DCFBC was the first ^18^F-labeled PSMA ligand which was clinically evaluated. It turned out that the ligand has a high plasma protein binding resulting in a persistent blood-pool activity. Further development led to the second notable ^18^F-labeled PSMA ligand [^18^F]DCFPyL, which has greater affinity for PSMA and fast(er) renal elimination providing better tumor-to-background ratios at early timepoints [4]. Another notable ligand is [^18^F]PSMA-1007 which was introduced in 2016 [7]. This radiopharmaceutical provides high tumor-to-background ratios while exhibiting a comparatively low urinary excretion. It was suggested that this qualifies the radiotracer as an excellent candidate for the diagnosis of recurrent PCa [4].

The exceptional imaging properties of PSMA-ligands are based on a high level of PSMA overexpression in case of PCa. The antigen itself is also expressed on several healthy tissues/organs and during several benign processes such as healing bone fracture, hemangiomas, or ganglia which can challenge possible diagnostic evaluation or discrimination of malignant processes [3, 8–13]. It was described that ^18^F-labeled PSMA ligands can have a higher rate of benign or unspecific tracer uptake as its ^68^Ga-labeled counterpart. This applies, for example, to the ganglia [14]. As small, morphologically diverse shaped structures such as roundish, oval, or tear-drop shaped bodies, ganglia of the sympathetic trunk can be easily misinterpreted as possible lymph node metastases [11]. In particular, the interpretation could be more challenging if such a structure showed a higher PSMA uptake in a follow-up study compared with the initial examination.

The aim of this retrospective analysis is to characterize, allocate, and quantify the extent of [^18^F]PSMA-1007 uptake in ganglia of the sympathetic trunk and to evaluate the intra-individual reproducibility of quantitative SUV values in these structures.

## Materials and Methods

### Patient Population

We retrospectively included 28 consecutive patients (median age 69 ± 9 years; range 49–90 years) with biochemical recurrence of PCa who underwent [^18^F]PSMA-1007 PET/CT in the Department of Nuclear Medicine of University Hospital Duesseldorf and, accordingly, a follow-up examination between August 2018 and August 2021. Studies with extensive lymph node metastases as well as studies without a follow-up examination were excluded.

### PET Image Acquisition

[^18^F]PSMA-1007 was synthesized as described previously [15] and injected via an intravenous bolus (baseline examination 235 ± 18 MBq; follow-up examination 232 ± 19 MBq). PET/CT acquisition was started 90–120 min after the injection of [^18^F]PSMA-1007 on a Siemens Biograph mCT (Siemens Healthineers). The examination included a diagnostic CT scan and the intravenous administration of a contrast agent at the beginning of the scan. The scans were acquired in 3D mode; the acquisition time per bed position was 3–4 min. Data were corrected for random, dead time, scatter, and attenuation. Iterative reconstruction of data using ordered-subsets expectation maximization algorithm (4 iterations, 8 subsets) was performed.

### Image Analysis

The analysis of the images was performed in consensus by two nuclear medicine physicians (D.S. and E.M.). The cervical, coeliac, and sacral ganglia were, preferably pairwise, identified on the iterative reconstructions (axial and MIP) and correlated with the CT. The [^18^F]PSMA-1007 uptake (SUV_max_/SUV_mean_), anatomical position in relation to adjacent vertebral bodies, and the side were assessed and noted both in baseline and follow-up scans. Criteria for ganglia were focal or tear-drop-shaped or ribbon-shaped [^18^F]PSMA-1007 uptake that could be correlated in CT in a typical localization para- or prevertebral.

### Statistical Analysis

We performed a two-way repeated measures ANOVA. The [^18^F]-PSMA-1007 PET/CT SUV metrics were defined as variables and the location of ganglia or the follow-up examination as factors. These statistical analyses were performed with SigmaPlot version 14.0 (Systat Software, Inc., San Jose, CA, USA). The quantitative data are demonstrated as mean ± SD. *P* values less than 0.001 were considered significant.

## Results

The [^18^F]PSMA-1007 uptake was detected in altogether 315 ganglia. The highest uptake was found in coeliac ganglia (SUV_max_ baseline 3.13 ± 0.85; in follow-up 3.11 ± 0.93) followed by cervical (SUV_max_ baseline 2.73 ± 0.69; in follow-up 2.67 ± 0.74) and sacral ganglia (SUV_max_ baseline 1.67 ± 0.50; in follow-up 1.64 ± 0.52). The SUV_mean_ was coeliac (baseline 2.28 ± 0.64; in follow-up 2.28 ± 0.66), cervical (baseline 1.62 ± 0.43; in follow-up 1.61 ± 0.43), and sacral (baseline 1.15 ± 0.33; in follow-up 1.12 ± 0.34) (Tables 1 and 2).

**Table 1:** Comparison of the developed SUV_max_ values in ganglia

Ganglia	Coeliac	Cervical	Sacral
	Baseline examination (SUV_max_)	Baseline examination (SUV_max_)	Baseline examination (SUV_max_)
Mean ± SD Max Min	3.13 ± 0.85 5.4 1.4	2.73 ± 0.69 4.4 1.4	1.66 ± 0.50 3.0 0.8
	Follow-up (SUV_max_)	Follow-up (SUV_max_)	Follow-up (SUV_max_)
Mean ± SD Max Min	3.11 ± 0.93 5.6 1.2	2.67 ± 0.74 4.4 1.2	1.64 ± 0.52 2.9 0.8

**Table 2:** Comparison of the developed SUV_mean_ values in ganglia

Ganglia	Coeliac	Cervical	Sacral
	Baseline examination (SUV_mean_)	Baseline examination (SUV_mean_)	Baseline examination (SUV_mean_)
Mean ± SD Max Min	2.28 ± 0.64 4.2 1.2	1.62 ± 0.43 2.9 1.0	1.15 ± 0.33 2.1 0.7
	Follow-up (SUV_mean_)	Follow-up (SUV_mean_)	Follow-up (SUV_mean_)
Mean ± SD Max Min	2.28 ± 0.66 3.6 1.0	1.61 ± 0.43 2.8 0.9	1.12 ± 0.34 2.0 0.6

**Fig. 1:**
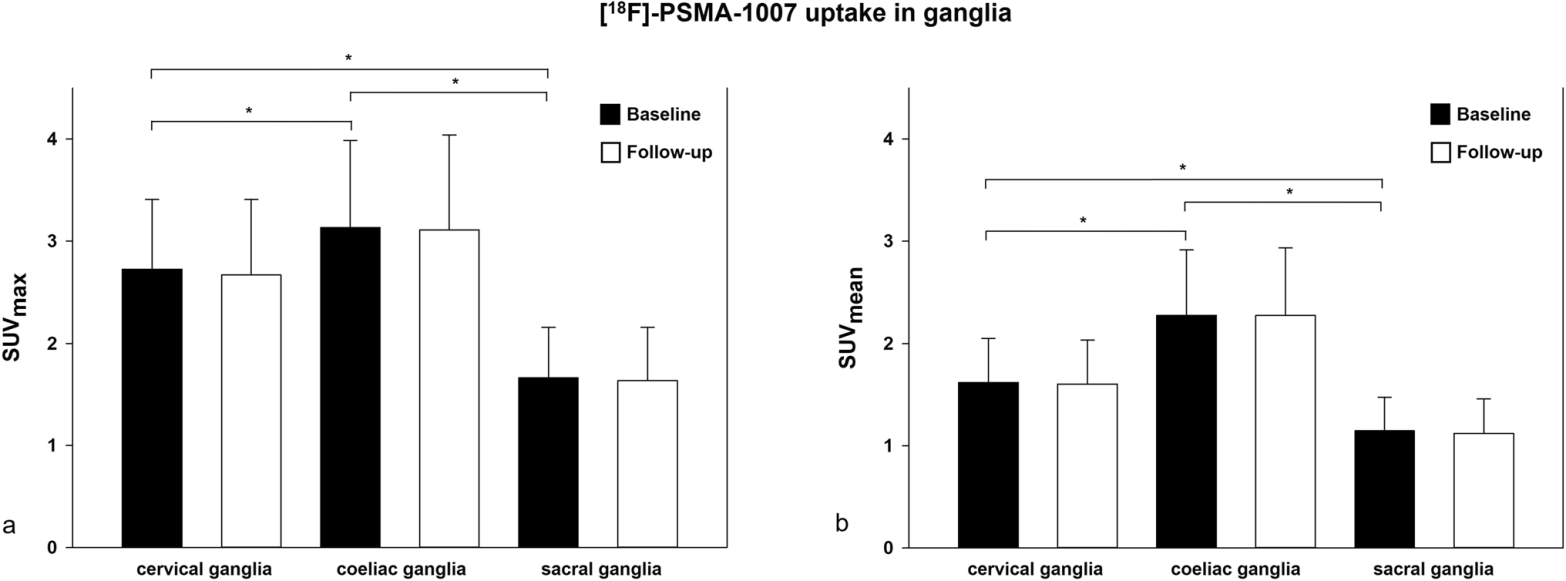
Comparison of [^18^F]-PSMA-1007 uptake in cervical, coeliac, and sacral ganglia in baseline examinations and follow-ups: **a** SUV_max_; **b** SUV_mean_; * significant differences.

There was a statistically significant difference of SUV_max_ and SUV_mean_ between all three anatomical locations of ganglia (*P* =  < 0.001) (Fig. 1).

**Fig. 2:**
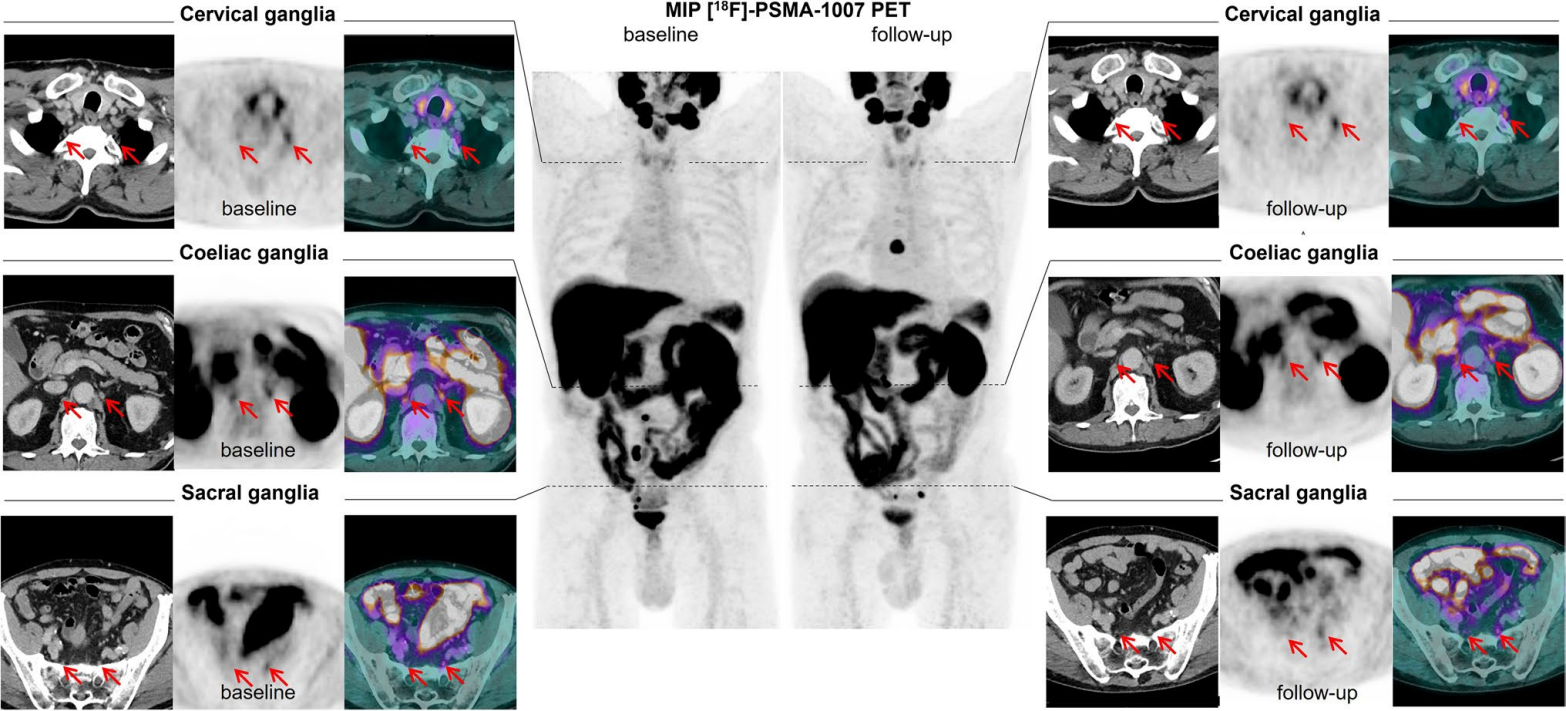
Comparison of ganglia of one patient in baseline and follow-up examinations in three anatomical localizations. Red lines show ganglia.

In a given ganglion station, there was no statistically significant difference of SUV_max_ or SUV_mean_ values between baseline and follow-up examination (P_SUVmax_ = 0,372; P_SUVmean_ = 0,627) (Fig. 2).

The localization of the ganglia was constant in baseline and follow-up scans in all cases. The cervical ganglia were found in the area of T1/T2, coeliac in the area of T12/L1, and sacral ganglia in the presacral region, respectively.

## Discussion

The physiological uptake of PSMA ligands in various normal tissues is an important pitfall in the PSMA-targeted imaging [2, 4, 16, 17]. One of these are ganglia of the sympathetic trunk. These small structures can have an increased uptake of PSMA ligands and mimic possible lymph node metastases [10, 11, 18, 19]. Rischpler et al. analyzed the [^68^Ga]Ga-PSMA-11 uptake in ganglia of the sympathetic trunk on a large cohort of patients. The uptake of the radiopharmaceutical in any ganglia was shown to be frequent and could be detected in 98.5% of patients. Ganglia can also be visualized with [^18^F]PSMA-1007 and could challenge the differentiation from malignant lesions [20].

In this retrospective study, we investigated the [^18^F]PSMA-1007 uptake in the ganglia of the sympathetic trunk and its intra-individual reproducibility.

We could clearly allocate and quantify a physiological [^18^F]PSMA-1007 ligand uptake in the ganglia in most cases. We determined no significant difference in intra-individual ^18^F-PSMA uptake in a given ganglion station between baseline and follow-up examination. However, significant difference of [^18^F]PSMA-1007 uptake was found between the three most common locations of ganglia. Highest [^68^Ga]Ga-PSMA-11 uptake in coeliac followed by cervical and sacral ganglia was reported [10]. This is in line with our findings with [^18^F]PSMA-1007. The mean SUV_max_ of coeliac and cervical ganglia in our study was a little bit higher than that of [^68^Ga]Ga-PSMA-11 by Rischpler et al. [10]. This higher SUV_max_ using [^18^F]PSMA-1007 can be attributed to the physical characteristics of ^18^F that allow an improved spatial resolution compared with ^68^Ga. In addition, [^18^F]PSMA-1007 has advantages in biodistribution. Its rapid blood clearance and high tumor-to-background ratios provide a high sensitivity and allow the detection of the smallest tumor lesions even in patients with low serum PSA levels. Furthermore, due to reduced urinary excretion of [^18^F]PSMA-1007, local recurrence of PCa or regional lymph node metastases can be more accurately detected [3, 4, 21–24]. But also, benign findings are detected more often. Especially unspecific uptake in ganglia, in the ribs, or in unspecific lymph nodes should not be misinterpreted as metastases [8, 21]. [^18^F]PSMA-1007 can have a higher rate of tracer uptake in ganglia compared to [^68^Ga]Ga-PSMA-11 [14]. This is of particular clinical importance in the assessment of the biochemical recurrence of PCa, because a differentiation between an oligometastatic state, which could be curable, and a metastatic disease should be done very carefully. If focal [^18^F]PSMA-1007 uptake in benign structures, such as ganglia, were misinterpreted as lymph node metastases, then an oligometastatic state could be incorrectly diagnosed as a metastatic disease with corresponding consequences for patients.

With the present study, we assessed SUV_max_ and SUV_mean_ values of [^18^F]PSMA-1007 in ganglia of the sympathetic trunk in typical anatomical localizations (coeliac, cervical, and sacral). These findings may contribute to an additional diagnostic certainty. However, individual coeliac and cervical ganglia had a relatively high SUV_max_ up to 5.6 and 4.4, respectively. This could cause a misinterpretation. But a typical anatomical allocation should support the correct differentiation of ganglia. Our study showed that detectable ganglia had the same anatomic localization in all cases at the beginning and in the follow-up. The cervical and coeliac ganglia were found along the vertebra in the area of T1/T2 and T12/L1, respectively. Sacral ganglia were accordingly localized in presacral region. In most cases, ganglia could be identified pairwise, which in addition to the typical anatomic localization and developed SUV_max_ values further facilitates the discrimination from metastases.

In the clinical routine, physicians can make a decision about malignancy on the basis of increasing uptake in subsequent examinations. To the best of our knowledge, our study is the first investigation analyzing physiological uptake of the ganglia using [^18^F]PSMA-1007 with very robust SUV_max_ values in intra-individual comparison. Consequently, an increased tracer uptake in a follow-up scan in the localization of hypothetic ganglia would be more likely a metastasis or an inflamed lymph node than a ganglion.

The limitations of this study are small patient cohort, lacking histopathological correlation and retrospective approach. Further studies with a larger patient population and preferably histopathological confirmation of unclear findings are needed.

## Conclusions

To our best knowledge, this is the first systematic description of the physiological [^18^F]PSMA-1007 uptake in ganglia of the sympathetic trunk. Hereby, we demonstrated that ganglia have a good intra-individual reproducibility of [^18^F]PSMA-1007 uptake in follow-up examinations as well as a low variability of SUV_max_ or SUV_mean_ and appear in the same, typical anatomical localization. These findings might improve and guide the differentiation of ganglia from malignant lesions in clinical practice.

## Data Availability

The data used and/or analyzed during the current study are available from the corresponding author on reasonable request.
